# KCTD10 as a selective cancer dependency from transcription-replication conflicts (TRCs)

**DOI:** 10.70401/acrt.2026.0015

**Published:** 2026-03-04

**Authors:** Jake A Kloeber, Bin Chen, Robert Mutter, Jinzhou Huang, Zhenkun Lou

**Affiliations:** 1 Division of Oncology Research, Mayo Clinic, Rochester, MN 55905, USA.; 2 Medical Scientist Training Program, Mayo Clinic, Rochester, MN 55905, USA.; 3 Department of Molecular Pharmacology and Experimental Therapeutics, Mayo Clinic, Rochester, MN 55905, USA.; 4 Department of Radiation Oncology, Mayo Clinic, Rochester, MN 55905, USA.

**Keywords:** DNA replication, transcription, transcription-replication conflicts, cancer therapy, KCTD10, genome stability

## Abstract

Transcription–replication conflicts (TRCs) are an increasingly recognized driver of genome instability in human cells. We recently identified the CUL3 adaptor KCTD10 as a sensor of co-directional TRCs, recruiting CUL3 to ubiquitinate transcriptional machinery and clear the path for replication forks. Here, we discuss the implications of this conflict-resolution pathway for human cancer. By integrating our mechanistic findings with large-scale functional genomics datasets, we identify oncogenic conditions that potentially create TRC-rich environments and render cells selectively dependent on KCTD10. These contexts reveal new mechanistic insights and potential therapeutic opportunities across a range of human cancers.

## 1. Introduction

Collisions between transcription and replication machineries are an unavoidable feature of eukaryotic genome duplication. In mammalian cells, replication forks advance at ~1–2 kb min^−1^ while RNA polymerase II elongates at comparable rates, making encounters inevitable when both traverse the same DNA template^[1,2]^. These conflicts occur in two orientations: head-on, in which the machineries approach from opposite directions, and co-directional, in which a replication fork overtakes a transcribing RNA polymerase. Although early biochemical studies suggested that co-directional collisions were relatively benign, emerging evidence indicates that they can produce substantial DNA damage, activate ATM-CHK2 signaling, and contribute to genome instability^[2–5]^. In particular, co-directional conflicts appear to be far more common in human cells than previously appreciated, owing to the strong alignment of replication and transcription programs across large genic regions.

Genome organization itself promotes co-directional encounters. Replication origins frequently overlap with transcription start sites, and ORC1 binds promoter-proximal RNAs in a manner that correlates with origin firing. Oncogenic processes profoundly disrupt the balance between transcription and replication. Hypertranscription, premature S-phase entry, metabolic bottlenecks, altered chromatin accessibility, and loss of checkpoint control all increase the frequency and severity of transcription-replication conflicts (TRCs)^[6]^. However, how cells detect and resolve co-directional conflicts has remained incompletely understood, and whether cancer cells with elevated TRC burdens might be selectively vulnerable to disruption of conflict-resolution pathways has not been systematically explored.

Genome-wide profiling approaches have provided direct evidence that TRCs accumulate in specific genomic and oncogenic contexts. R-loop mapping and nascent transcription assays reveal that highly transcribed genes, amplified loci, and regions of altered chromatin organization are enriched for transcription-associated replication stress^[4–6]^. Recent studies further demonstrate that extrachromosomal DNA (ecDNA) and amplified genomic regions, often containing oncogenes, exhibit pervasive transcription and replication alterations, creating conflict-prone genomic domains^[7]^. Together, these datasets support the concept that oncogenic states characterized by hypertranscription, altered replication timing, or aberrant genome architecture generate TRC-rich environments, providing a framework for considering selective dependency on conflict-resolution factors.

Our recent study established that KCTD10 is required for the resolution of endogenous co-directional TRCs in human cells^[3]^. In genome-wide profiling, KCTD10 was found to be enriched at genomic sites associated with co-directional TRCs, and KCTD10-deficient cells accumulate DNA damage markers specifically when replication and transcription proceed in the same direction, as assessed using orientation-specific reporter constructs. Furthermore, KCTD10-deficient cells accumulate γH2AX foci, spontaneous micronuclei, and elevated CHK2 phosphorylation, hallmarks of damage associated with co-directional collisions. Mechanistically, KCTD10 binds both PCNA at the replication fork and components of the RNAPII complex. Upon encountering a transcription complex, KCTD10 self-associates into higher-order assemblies that create an interface for CUL3 recruitment. The resulting CUL3-KCTD10 complex ubiquitinates TFIIS/TCEA2, promoting clearance of the transcription machinery and allowing the fork to proceed. In the absence of KCTD10, TCEA2 persists on chromatin while trying to restart RNAPII, RNAPII remains obstructive, and conflicts accumulate. These features define KCTD10 as a bifunctional molecular bridge linking replication and transcription, and as a gatekeeper for conflict resolution. This raises the possibility that tumors with heightened co-directional TRCs may acquire a strong selective dependency on KCTD10.

Here, we examine how this conflict-resolution pathway intersects with oncogenic states and discuss potential mechanistic links. Integrating our findings with cancer dependency mapping and transcriptional profiling reveals several oncogenic contexts in which co-directional TRCs are potentially pervasive, and KCTD10 becomes selectively essential.

## 2. Identifying Selective KCTD10 Co-Dependencies in Human Cancer

**Figure 1.**
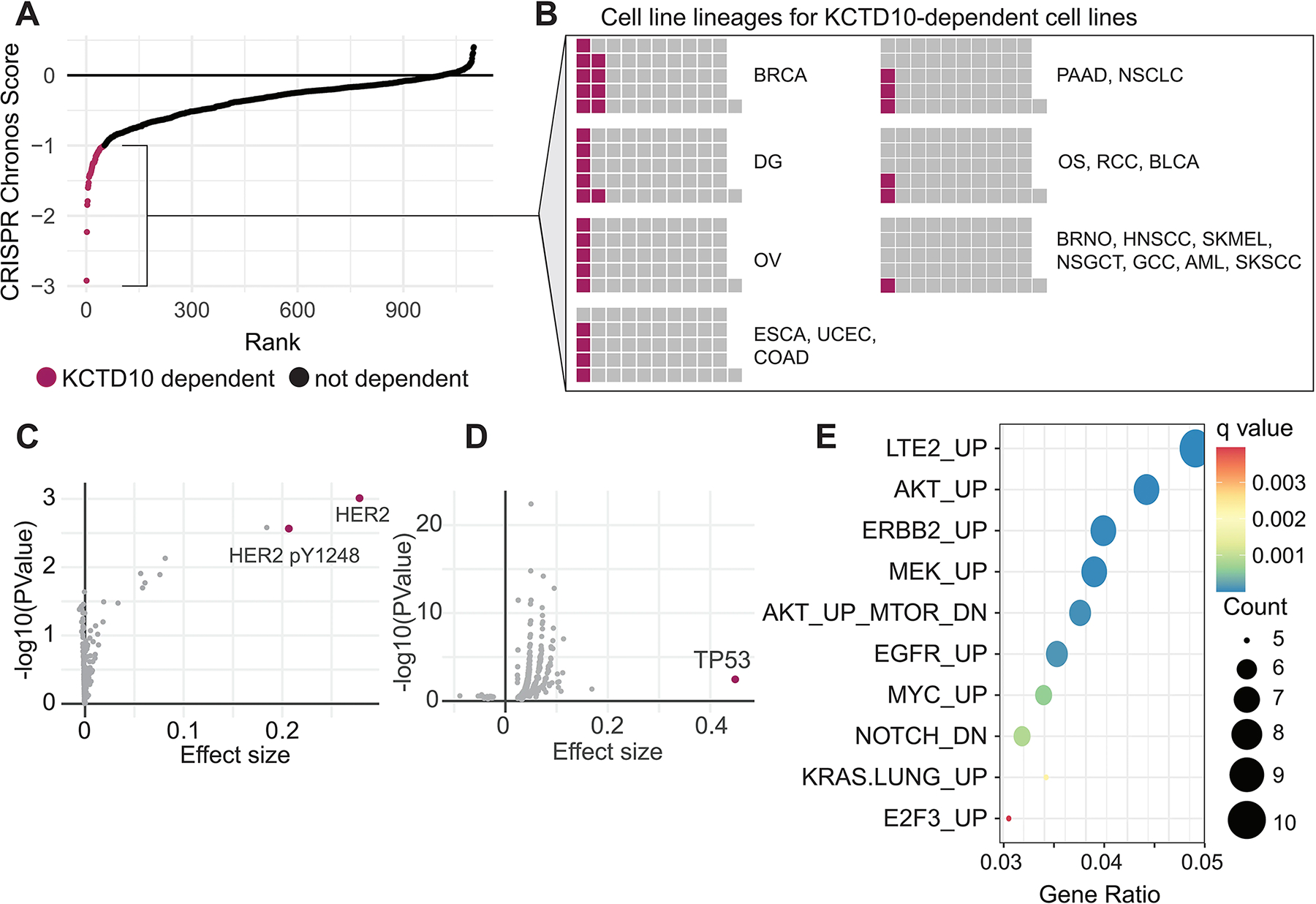
Characterization of KCTD10 dependency across human cancer cell lines. (A) CRISPR dependency scores for KCTD10. Cell lines with a Chronos score < −1 are considered dependent (magenta); (B) Cell line lineages for KCTD10-dependent cell lines. Multiple listed cancers indicate the same proportion for each of the cancers; (C) Enriched proteins in KCTD10-dependent cancer cell lines relative to all other cancer cell lines; (D) Enriched damaging mutations in KCTD10-dependent cancer cell lines relative to all other cancer cell lines; (E) Gene set enrichment analysis of oncogenic pathways in upregulated genes among TCGA samples that share a transcriptional profile with KCTD10-dependent cell lines using Celligner^[8]^. BRCA: breast invasive carcinoma; DG: diffuse glioma; OV: ovarian epithelial tumor; ESCA: esophageal squamous cell carcinoma; UCEC: uterine corpus endometrial carcinoma; COAD: colon adenocarcinoma; PAAD: pancreatic adenocarcinoma; NSCLC: non-small cell lung cancer; OS: osteosarcoma; RCC: renal cell carcinoma; BLCA: bladder carcinoma; BRNO: breast neoplasm, not otherwise specified; HNSCC: head and neck squamous cell carcinoma; SKMEL: melanoma; NSGCT: non-seminominous germ cell tumor; GCC: glassy cell carcinoma; AML: acute myeloid leukemia; SKSCC: cutaneous squamous cell carcinoma.

Based on our work showing that KCTD10 resolves co-directional conflicts between DNA replication and transcription, we hypothesized that KCTD10 may be an essential gene for cells with specific transcriptional programs. In the cancer dependency map (depmap.org), KCTD10 dependency was seen in 51 cell lines (out of 1,100 cell lines tested) (Figure 1A). Out of all these cell lines, the most enriched subtype was breast invasive carcinoma cell lines, followed by diffuse glioma and then ovarian epithelial tumor cell lines (Figure 1B). The most prevalent signature among KCTD10 dependent cell lines was enrichment of ERBB2 (HER2) protein and activated HER2 (pY1248), and damaging mutations in the *TP53* tumor suppressor (Figure 1C,D). To further identify transcriptional features in these KCTD10-dependent cell lines, we utilized an unsupervised alignment method (Celligner)^[8]^ to identify their nearest neighbors among samples from the cancer genome atlas (TCGA). Gene set enrichment analysis of RNAseq data from these TCGA samples revealed upregulation of several oncogenic pathways, including pathways associated with AKT, ERBB2, MYC, and MEK signaling compared to all other breast cancer samples (Figure 1E), supporting a broader transcriptional environment that may promote co-directional TRCs.

The 5% dependency rate observed here is comparable to other clinically validated cancer-specific vulnerabilities, such as *BRCA1/2* alterations, which occur in approximately 15–20% of ovarian cancers and have supported successful targeted therapeutic strategies. Notably, *BRCA1* itself is classified as a dependency in a comparable fraction of cancer cell lines in DepMap (~3.8%), illustrating that even relatively infrequent genetic dependencies in large-scale screening datasets can nonetheless reflect biologically and clinically meaningful vulnerabilities. Moreover, because DepMap screens are performed under standard *in vitro* culture conditions, they may underestimate dependencies that become critical under *in vivo* tumor constraints, including hypoxia, nutrient limitation, or therapeutic pressure. Together, these considerations suggest that, if mechanistically validated, KCTD10 inhibition could be therapeutically relevant for a defined subset of patients rather than representing a rare outlier, and support interpreting KCTD10 dependency as a context-specific vulnerability.

The observation that most cancer cell lines are not dependent on KCTD10, even those harboring TRC-promoting alterations, likely reflects the availability of alternative conflict-resolution mechanisms. Multiple pathways can resolve or mitigate TRCs independently of KCTD10, including RECQ5-mediated displacement of RNAPII from stalled forks, activation of the Fanconi anemia pathway, and fork remodeling by SMARCAL1, ZRANB3, and HLTF at sites of TRCs^[4]^. Many of these mechanisms have been implicated in resolving R-loop–associated conflicts, whereas KCTD10 appears to function in co-directional TRCs independent of R-loops^[3]^. Consistent with this latter point, recent work shows that CFAP20 plays a similar role to KCTD10 in resolving co-directional transcription–replication conflicts at R-loop–prone regions^[9]^, further underscoring that distinct molecular factors can independently alleviate co-directional stress. In addition, DNA damage caused by KCTD10 loss can also be repaired if cells have enhanced DNA repair capabilities. Cells with lower transcriptional output or fewer intragenic replication origins may also experience fewer co-directional conflicts, reducing selective pressure on KCTD10. KCTD10 dependency likely emerges when co-directional TRC burden exceeds the collective capacity of these alternative pathways, a threshold that may be reached only when multiple TRC-promoting alterations converge in the same cell.

Taken together, these data suggest several promising avenues for targeting KCTD10 in specific oncogenic contexts. We will explore these briefly below and highlight potential mechanistic explanations for these relationships, with an emphasis on how distinct oncogenic states may shape KCTD10 dependency.

### 2.1 Oncogene amplification

Amplification of oncogenes such as *MYC*, *CCNE1*, *HER2*, and *EGFR* is commonly seen across several cancers and has a wide range of impacts that could confer KCTD10 dependence. For example, amplified *MYC* and *CCNE1* drive premature S-phase entry by shortening the G1 phase of the cell cycle. This abbreviated G1 phase prevents complete transcription of genic regions before replication initiates, leading to the firing of ectopic replication origins within actively transcribed genes^[10–12]^. These intragenic origins create frequent collisions between the replisome and transcription machinery. The consequences of cyclin E1 overexpression have been further characterized in studies showing that deregulated cyclin E causes cells to enter mitosis with short, unreplicated genomic segments at specific loci, particularly at late-replicating domains and fragile sites, leading to deletions at these regions^[13,14]^. Such perturbations are expected to increase the burden of co-directional TRCs that may require KCTD10 for efficient resolution.

Replication timing and origin organization are likely key determinants of where co-directional TRCs arise. Indeed, high-resolution break mapping has revealed that DNA double-strand breaks from TRCs preferentially occur at timing transition regions where sparse replication origins generate long-traveling unidirectional forks^[15]^. Shortened G1 phases driven by oncogenic signaling promote intragenic origin firing and increase origin proximity to actively transcribed promoters. When combined with replication fork slowing or compensatory dormant origin activation, these features extend the temporal overlap between replication and transcription. Consistent with this model, accelerated G1-to-S phase progression driven by PPM1D gain-of-function (GOF) has been shown to impair origin licensing and exacerbate TRCs and fork slowing in CCNE1-overexpressing cells^[16]^. As a result, replication forks are more likely to encounter transcription complexes in a co-directional orientation at highly expressed loci, creating genomic contexts in which KCTD10-mediated conflict resolution may become particularly important.

MYC not only shortens G1 but also increases the overall transcriptional output of the cell, creating a cellular environment where co-directional conflicts are more prone to occur. Several studies have demonstrated that MYC functions as a “transcriptional amplifier” in some contexts, accumulating at promoters of already active genes and amplifying their transcriptional output rather than activating discrete new target genes^[17]^. However, the biology of MYC is considerably more complex, and recent work has revealed that MYC exhibits both context-independent amplification and context-dependent gene-specific regulation^[18]^. In oncogenic settings, elevated MYC levels enable occupancy of low-affinity binding sites at enhancers in addition to high-affinity promoter sites, leading to selective activation and repression of specific gene programs that vary between tumor types^[19–21]^. This dual nature helps explain MYC’s diverse effects across different cellular contexts and its potent oncogenic capacity when deregulated. Notably, the relationship between hypertranscription and replication stress extends to multiple oncogenic pathways beyond MYC activation, and even certain targeted cancer therapies can promote global increases in transcription leading to R-loop accumulation and TRCs^[6]^. These MYC-driven alterations therefore provide a plausible mechanistic basis for increased KCTD10 dependency in MYC-amplified tumors.

More recently, MYC proteins have also been shown to play multiple roles in managing the genomic stress generated by high transcriptional output. For example, MYC nucleates a “topoisome” complex that unites topoisomerase 1 (TOP1) with either TOP2A (in MYC-expressing cells) or TOP2B (in MYCN-expressing cells)^[22]^. Beyond simply recruiting these topoisomerases to sites of transcription, MYC directly stimulates their enzymatic activities at promoters, gene bodies, and enhancers to resolve torsional stress that would otherwise impede transcription and replication. Additionally, when transcription elongation, mRNA splicing, or proteasome function is perturbed, MYC dissociates from promoters and forms multimeric, often sphere-like structures that accumulate on chromatin adjacent to stalled replication forks^[23]^. These MYC multimers surround DNA repair proteins including FANCD2, ATR, and BRCA1, effectively shielding replication forks from collisions with RNA polymerase II. At active promoters, MYC multimers block antisense transcription and stabilize FANCD2 association with chromatin, limiting DNA double-strand break formation during S-phase^[23]^. Together, these mechanisms become overwhelmed when MYC is deregulated, contributing to the genomic instability characteristic of MYC-driven cancers^[24]^, and may contribute to KCTD10 dependency. In such scenarios, KCTD10 likely acts in parallel with MYC-mediated stress responses to safeguard replication forks at co-directional collision sites.

Oncogene amplification can also create aberrant genome architecture prone to conflicts. Focal amplifications are frequently mediated by the generation of ecDNA, circular DNA elements lacking centromeres that are found in approximately 17% of tumors across cancer types and are particularly enriched in aggressive malignancies, including glioblastoma and sarcomas^[25,26]^. The circular topology and highly accessible chromatin of ecDNA drives pervasive transcription not only of the amplified oncogene but also of non-coding, antisense, and intergenic regions across the entire element^[7]^. Genome-wide analyses have shown that these transcriptionally dense ecDNA domains are particularly susceptible to TRCs, making them enriched sites of replication stress. Importantly, ecDNA exists in dynamic equilibrium with intrachromosomal amplification structures. Homogeneously staining regions (HSRs) can arise through breakage-fusion-bridge cycles that generate amplicons with characteristic inverted duplication junctions. However, ecDNA and HSRs are mechanistically interconvertible, as ecDNA has been shown to reintegrate into chromosomes to form HSRs, while HSRs trapped in anaphase bridges can undergo chromothripsis to regenerate ecDNA^[27,28]^. Regardless of their structural state, these amplified regions share features that may increase the probability of collisions between replication and transcription machinery, creating an environment highly susceptible to TRCs^[7]^, and potentially heightening reliance on KCTD10 at these conflict-prone loci.

The *HER2*/*ERBB2* amplicon presents a particularly compelling case for KCTD10 dependency. HER2-amplified breast cancers frequently harbor complex amplicon structures on chromosome 17q12, with a core region spanning 85 kb to over 1.8 Mb that includes not only *HER2* but also co-amplified genes such as *GRB7*, *TOP2A*, *STARD3*, and others^[29]^. These amplicons often display “firestorm” patterns of clustered amplification peaks and complex chromosomal rearrangements^[29,30]^. A significant proportion of HER2-positive breast cancers are found to harbor *HER2* amplification on ecDNA. HER2 amplification and PTEN loss have been shown to promote DNA replication stress and activate APOBEC3 mutagenesis via an ATR/Chk1-dependent pathway, with HER2-enriched breast carcinomas displaying elevated levels of replication stress-associated DNA damage *in vivo*^[31]^. Additionally, the constitutive activation of downstream MAPK and PI3K signaling pathways drives robust transcriptional programs that could increase the probability of TRCs encounters in HER2-amplified cells. This is consistent with the strong KCTD10 dependency signal observed in HER2-amplified breast cancer cell lines in DepMap analyses.

EGFR amplification, particularly prevalent in glioblastoma, creates a similar dependency through sustained receptor tyrosine kinase signaling. In glioblastoma, EGFR amplification frequently occurs on ecDNA, with distinct ecDNA species containing either wild-type EGFR or variant forms such as EGFRvIII^[32]^. Spatiotemporal modeling of ecDNA evolution in glioblastoma has revealed that *EGFR*-containing ecDNAs reach very high copy numbers (mean of 50 copies per cell) and are under strong positive selection^[33]^. The co-amplification of EGFR with MYC in many gliomas compounds the transcriptional burden and TRC frequency. Our analysis of KCTD10-dependent cell lines in DepMap reveals that glioblastoma cell lines with EGFR amplification are significantly enriched among KCTD10-dependent tumors. These observations, together support a model in which ecDNA-based EGFR amplification not only drives oncogenic signaling but also shapes TRC-rich genomic domains that depend on KCTD10 for conflict resolution.

### 2.2 Constitutive pathway activation

While oncogene amplification can dramatically upregulate signaling, activating mutations in signaling proteins such as BRAF, KRAS, and PIK3CA lock downstream pathways in a perpetually active state. These events represent another major route to KCTD10 dependency by driving sustained transcriptional programs that increase TRC frequency.

The BRAF V600E mutation, found in approximately 50% of melanomas and a significant fraction of colorectal and thyroid cancers, provides a paradigmatic example. This mutation renders BRAF constitutively active, leading to sustained ERK signaling independent of upstream growth factor input. BRAF V600E colorectal cells appear to be more prone to forming TRCs, which confers dependency on the splicing factor SFPQ for proper DNA replication^[34]^. In melanoma, activated ERK drives the microphthalmia-associated transcription factor (MITF) transcription factor through BRN2 (N-Oct3), which orchestrates a lineage-specific transcriptional program essential for melanocyte identity and survival^[35]^. MITF-expressing melanoma cells accumulate stalled replication forks and display defects in homologous recombination-mediated repair, and high MITF levels correlate with increased single-nucleotide variant burden in melanoma samples^[36]^. The high transcriptional output at MITF target genes may create hotspots for TRCs, particularly at highly expressed pigmentation genes and cell cycle regulators. Notably, high MITF expression has been linked with acquired resistance to BRAF inhibitors^[37]^, which could represent accelerated clonal evolution secondary to these conflicts. These MITF-associated transcriptional features may elevate reliance on KCTD10-mediated resolution of co-directional TRCs.

KRAS mutations, prevalent in pancreatic, colorectal, and lung adenocarcinomas, may similarly drive KCTD10 dependency through constitutive activation of multiple downstream effector pathways, including MAPK, PI3K, and RAL-GEF signaling cascades. RAS overexpression causes increased global transcriptional activity through elevated expression of TATA-binding protein and other components of the general transcription machinery^[38]^. Elevated transcription results in replication fork slowing and DNA damage. Notably, recent work has demonstrated that TRCs are enriched in PDAC compared to other solid tumors, that this enrichment is KRAS-dependent. Further, a PCNA-targeting compound that enhances RNAPII-PCNA interactions exhibited dose-dependent cytotoxicity that was reliant on KRAS G12D expression^[39]^. The BER pathway appears to regulate TRC levels in PDAC, as its inhibition traps RNAPII at nascent DNA and activates the ATR-CHK1 checkpoint^[40]^. When combined with RAS-mediated acceleration of cell cycle progression, there are abundant opportunities for TRCs to arise across the genome.

KRAS-mutant cells also show altered metabolic dependencies that can compromise S-phase progression. KRAS/LKB1-mutant lung cancer cells depend on CPS1 for pyrimidine biosynthesis, and nucleotide depletion in these cells causes replicative stress and DNA polymerase stalling^[41]^. Similarly, KRAS-driven cancer cells that are deprived of glutamine arrest in S-phase due to ATR activation and deoxynucleotide depletion^[42]^. Critically, nucleotide insufficiency slows replication fork progression, which extends the window during which forks traverse transcriptionally active regions and triggers compensatory firing of dormant replication origins, events that increase the frequency of TRCs genome wide. Thus, KRAS-mutant cells face a compounded threat from oncogene-driven transcriptional hyperactivity combined with metabolically compromised replication, creating widespread opportunities for conflicts that would necessitate KCTD10-mediated resolution. These combined metabolic and transcriptional effects provide additional rationale for KRAS-associated KCTD10 dependency.

The PI3K-AKT-mTOR axis represents a third pathway through which constitutive activation may generate KCTD10 dependency. Activating mutations in PIK3CA or loss of the PTEN tumor suppressor result in sustained mTOR signaling, which drives high-level transcription of metabolic genes, ribosomal biogenesis components, and other targets of the mTORC1 complex. The ribosomal DNA loci, which are among the most heavily transcribed regions in the genome, represent known hotspots for TRCs that require specialized helicases such as RECQ5 for resolution^[43]^. Sustained mTORC1 activation amplifies rDNA transcription and may therefore increase the frequency and severity of these inherently conflict-prone loci. More broadly, the high transcriptional demands of mTOR-activated cells across both rDNA and mTORC1 target genes increase the likelihood of collisions between replication and transcription machinery genome-wide.

Beyond its transcriptional effects, the PI3K-AKT axis directly influences replication fork dynamics. PTEN, independent of its canonical lipid phosphatase activity, dephosphorylates the replicative helicase component MCM2 and restricts replication fork progression under conditions of replication stress; PTEN-deficient cells exhibit unrestrained fork progression and accumulate chromosomal aberrations^[44]^. mTORC1 signaling also promotes de novo pyrimidine synthesis through S6K1-mediated phosphorylation of CAD, coupling growth signaling to nucleotide production^[45]^. Paradoxically, while this ensures nucleotide supply during rapid proliferation, it may also create metabolic inflexibility, where PI3K/mTOR-hyperactive cells become dependent on this pathway for adequate nucleotide pools, and any disruption could precipitate replication stress. Such metabolic-replicative constraints may increase KCTD10 relevance in maintaining fork progression.

### 2.3 SWI/SNF dysfunction

Inactivating mutations in SWI/SNF chromatin remodeling complex subunits, particularly ARID1A, represent a distinct mechanism of KCTD10 dependency that operates through chromatin accessibility defects rather than transcriptional amplification. SWI/SNF complexes utilize ATP hydrolysis to mobilize nucleosomes, and their dysfunction compromises the proper organization of chromatin during both transcription and DNA replication. Components of SWI/SNF are mutated in approximately 20% of all human cancers, collectively establishing them among the most frequently altered chromatin regulators in malignancies^[46]^. In particular, ARID1A is one of the most frequently mutated genes, with loss-of-function mutations occurring in approximately 50% of ovarian clear cell carcinomas, 30% of endometrioid carcinomas, and significant fractions of gastric, bladder, and hepatocellular carcinomas^[47]^. ARID1A loss results in accumulated DNA base lesions, increased abasic sites, and delayed recruitment of base excision repair effectors^[48]^. The combination of temozolomide with PARP inhibitors potently elicits double-strand DNA breaks, replication stress, and replication fork instability specifically in ARID1A-deficient cells, suggesting that these cells possess underlying vulnerabilities in handling replication-associated DNA damage.

The SMARCA4/BRG1 ATPase subunit is also frequently mutated in cancer, particularly in small cell carcinomas of the ovary hypercalcemic type, SMARCA4-deficient thoracic sarcomas, and subsets of lung and bladder carcinomas^[49]^. Loss of SMARCB1/SNF5, another core SWI/SNF subunit, drives aggressive rhabdoid tumors in children^[50]^. Importantly, SNF5 antagonizes MYC activity by inhibiting its DNA-binding ability, and loss of SNF5 leads to activation of MYC target genes^[51]^. This illustrates how SWI/SNF loss can indirectly promote TRC formation by derepressing oncogenic transcription factors, converging with the transcriptional mechanisms described above, and thereby creating additional situations where KCTD10 activity becomes relevant.

The SWI/SNF complex is also tightly linked with the function of TOP2A. Endogenous BAF complexes interact directly with endogenous TOP2A through ARID1A and are required for the binding of TOP2A to approximately 12,000 sites across the genome^[52]^. TOP2A chromatin binding is dependent on the ATPase activity of BRG1, which is compromised in oncogenic BRG1 mutants. Deletion of *Brg1* in mouse cells, as well as expression of BRG1 point mutants identified in human tumors, leads to anaphase bridge formation and a G2/M-phase block characteristic of the decatenation checkpoint. In ARID1A-deficient cells, defective TOP2A localization would be expected to cause accumulation of topological stress that impedes replication fork progression and exacerbates TRCs, further increasing the requirement for auxiliary TRC-resolution mechanisms such as KCTD10.

We propose that the KCTD10-dependence seen in ovarian cancer cells may be due to their underlying SWI/SNF complex deficiency, fundamentally compromising the chromatin environment for efficient conflict resolution. Without proper SWI/SNF-dependent TOP2A localization and appropriate checkpoint signaling, cells accumulate unresolved TRCs that overwhelm canonical resolution pathways. KCTD10’s ability to bridge the replisome and transcription machinery and facilitate RNAPII removal provides an alternative resolution mechanism that becomes essential in this context, supporting a model in which SWI/SNF dysfunction sensitizes cells to KCTD10 loss.

### 2.4 TP53 aberrations

Mutations in or loss of the tumor suppressor TP53 may also establish KCTD10 dependency through multiple potential mechanisms. *TP53* is the most frequently mutated gene in human cancer, with alterations occurring in approximately 50% of all malignancies^[53]^. Critically, *TP53* aberrations comprise two functionally distinct categories: loss-of-function mutations that eliminate p53 tumor suppressor activity, and missense mutations that can confer GOF properties. Both modes of p53 dysfunction may contribute to TRCs through different mechanisms, potentially increasing reliance on TRC-resolution factors such as KCTD10.

The p53 protein serves as a critical coordinator of transcription and DNA replication timing, ensuring that cells do not enter S-phase with unresolved conflicts or damage. In its absence, cells lose the G1/S checkpoint that would normally pause the cell cycle in response to replication stress signals. A key consequence of TP53 loss is deregulated E2F transcriptional activity. Under normal conditions, p53 induces p21 (CDKN1A), which inhibits cyclin-dependent kinases and maintains the retinoblastoma protein (RB) in its active, E2F-repressing state. Additionally, E2F7 is a direct transcriptional target of p53 that cooperates with RB to promote cell cycle arrest; E2F7 binds and represses E2F target genes, and its loss abrogates p53-dependent repression of proliferation-associated genes^[54,55]^. When p53 is lost, E2F7 is no longer induced, and E2F target genes become derepressed, driving unscheduled S-phase entry. Notably, our KCTD10 CUT & RUN analysis identified binding sites in E2F target genes that were enriched by inducing TRCs with the TOP2 inhibitor ICRF193^[3]^, suggesting these may be sites of co-directional TRCs, and potentially points where KCTD10 function becomes increasingly important.

p53 loss also enables tolerance of replication stress-inducing genomic aberrations. In ATRX-deficient neuroblastoma cells, loss of p53 suppresses replication stress-induced DNA damage by abrogating cell cycle arrest and reducing G-quadruplex accumulation^[56]^. Furthermore, p53 deficiency induces expression of G4 DNA helicases and FANCD2, suggesting that p53 loss attenuates replication stress by upregulating compensatory DNA repair mechanisms and stabilizing replication fork integrity. This permissive environment allows cells with chromatin remodeling defects to evade checkpoint activation and continue proliferating despite ongoing replication stress. Interestingly, KCTD10 loss reduces the cytotoxic effects of the G4 stabilizer pyridostatin^[3,57]^, suggesting a functional connection between KCTD10 and the resolution of G4-associated replication barriers, which may further reinforce KCTD10 dependency in p53-deficient contexts.

Distinct from simple loss-of-function, the majority of p53 mutations in human cancer are missense mutations that result in expression of stable, full-length mutant proteins^[53,58]^. These GOF mutants frequently acquire novel oncogenic activities that represent active drivers of tumor progression. Different missense mutations confer unique activities, with “hotspot” mutations (R175H, R248W, R273H, R249S) particularly well-characterized for their GOF properties^[59]^. Mutant p53 GOF activities are primarily mediated through interactions with other transcription factors and cellular effectors that alter gene expression programs^[59]^. Mutant p53 has been shown to interact with and co-opt transcription factors including NF-Y, SREBPs, ETS2, NF-κB, and members of the p63/p73 family^[53]^. These interactions can dramatically alter tumor cell behavior by driving expression of genes that promote proliferation, invasion, metastasis, and drug resistance. Importantly, the specific transcription factor partners available to mutant p53 are context-dependent, varying with cell type, disease state, and microenvironmental conditions, which may influence how mutant p53 shapes TRC landscapes that intersect with KCTD10 function.

Mutant p53 GOF also impacts metabolic reprogramming that may exacerbate TRCs. Wild-type p53 normally suppresses aerobic glycolysis and promotes oxidative phosphorylation, thereby restraining the Warburg effect^[60]^. Mutant p53, by contrast, promotes aerobic glycolysis through multiple mechanisms, including the activation of the mevalonate pathway via SREBP binding and enhanced glucose uptake through RhoA/ROCK-mediated GLUT1 membrane translocation^[53,61]^. This metabolic shift supports the selfish phenotype of rapid proliferation but may also create imbalances between biosynthetic capacity and replication demands. The resulting metabolic inflexibility, coupled with the transcriptional programs driven by mutant p53 interactions with NF-κB, HIF-1α, and other factors, creates conditions that favor TRCs by simultaneously increasing transcriptional output and accelerating cell cycle progression, thereby potentially enhancing KCTD10 relevance in managing co-directional conflicts.

The therapeutic implications of mutant p53 GOF are distinct from those of p53 loss. Because stabilization of mutant p53 protein is crucial for its oncogenic activities, strategies to deplete mutant p53 or restore the wild-type conformation have attracted significant interest^[62,63]^. However, the question of whether targeting mutant p53 provides therapeutic benefit remains an area of active investigation, as GOF may be more or less relevant depending on tumor context and the specific mutation involved. As an alternative, KCTD10 may provide an alternative means to target some TP53 mutant cancers, particularly those in which TRCs contribute substantially to cellular fitness.

Both p53 loss and GOF mutations ultimately create conditions that increase TRC frequency. Loss of p53 removes G1/S checkpoint control, permits unscheduled S-phase entry with ongoing transcription, and allows tolerance of replication stress that would otherwise trigger cell cycle arrest. GOF mutations compound these effects by actively driving transcriptional programs and metabolic adaptations that increase biosynthetic demands. When p53 dysfunction co-occurs with other TRC-promoting alterations, the burden of conflicts would be expected to increase further. In such contexts, CUL3-KCTD10’s function may become essential for cellular survival, establishing a dependency that could be therapeutically exploited across diverse TP53-altered cancers, supporting the model that p53 dysregulation sensitizes cells to KCTD10 loss.

## 3. Limitations

While DepMap provides a valuable resource for identifying cancer dependencies, several important limitations must be considered when interpreting these data. First, dependency scores reflect fitness defects under standard *in vitro* culture conditions and may not capture dependencies that emerge under specific selective constraints, including metabolic and therapeutic pressures that characterize the *in vivo* tumor environment. Second, CRISPR-Cas9 screens can exhibit guide-specific off-target effects and variable cutting efficiency across different genomic contexts. Third, correlations between dependency and genomic features are inherently associative and do not establish causality. Fourth, cancer cell line models, while useful, may not fully recapitulate the complex tumor microenvironment, stromal interactions, or immune surveillance present in patient tumors. Therefore, the associations we describe should be viewed as hypothesis-generating insights that prioritize contexts for experimental validation rather than definitive proof of mechanism.

## 4. Conclusions

We propose that KCTD10 is selectively essential in cancer cells prone to forming co-directional TRCs. These conflicts arise through multiple potential mechanisms: (1) oncogene amplification that drives hypertranscription and aberrant genome architecture; (2) constitutive activation of MAPK, PI3K, and other signaling pathways that sustain transcriptional programs; (3) SWI/SNF dysfunction that compromises chromatin organization and TOP2A-mediated topological resolution; and (4) TP53 aberrations that remove checkpoint control and, in the case of GOF mutations, actively drive oncogenic transcription. While these mechanisms differ in their molecular details, they converge on a common phenotype: elevated frequency and/or severity of co-directional conflicts that overwhelm the cell’s resolution capacity in the absence of KCTD10, thereby creating conditions in which KCTD10 becomes selectively essential.

Importantly, these pathways frequently intersect in human tumors. HER2-amplified breast cancers, which emerged as the most enriched lineage in our dependency analysis, exemplify this by combining gene amplification with constitutive activation of both MAPK and PI3K signaling. Similar convergence occurs in KRAS-mutant pancreatic cancers with concurrent TP53 loss, or in ovarian clear cell carcinomas harboring both ARID1A and PIK3CA mutations, indicating that KCTD10 dependency often arises from the combined effects of multiple TRC-promoting alterations.

The identification of KCTD10 as a selective dependency in these contexts has significant therapeutic implications. Unlike general replication stress-inducing agents, which harm rapidly dividing normal tissues, targeting CUL3-KCTD10 could selectively eliminate cancer cells harboring specific oncogenic alterations while sparing normal cells with lower TRC burdens. However, the mechanistic framework presented here remains to be empirically validated, and the therapeutic window for CUL3-KCTD10 inhibition in normal tissues is unknown, highlighting the need for further functional and preclinical studies.

## 5. Future Directions

The framework presented here generates testable predictions that should be empirically validated. KCTD10 dependency should be confirmed across additional cell lines representing each oncogenic context, with particular attention to HER2-amplified breast cancers given their prominence in our DepMap analysis. Patient-derived xenografts and genetically engineered mouse models will be essential to establish whether KCTD10 inhibition is therapeutically viable. Of particular importance is determining the therapeutic window, as normal proliferating tissues also experience TRCs, and the degree to which they depend on KCTD10 remains unknown.

Mechanistic studies should address whether KCTD10 responds uniformly to all co-directional conflicts or whether specific genomic features, such as transcriptional output, gene length, origin proximity, or chromatin accessibility, create privileged sites of KCTD10 action. High-resolution conflict-mapping approaches in KCTD10-proficient and -deficient cells could identify the loci and structural features that render conflicts KCTD10-dependent.

The CUL3-KCTD10 complex presents multiple potential intervention points, including disruption of KCTD10-PCNA or KCTD10-RNAPII interactions, blockade of oligomerization, or inhibition of CUL3-mediated ubiquitination. Structural characterization of KCTD10’s BTB domain and its binding interfaces will be critical for identifying druggable surfaces. Proteolysis-targeting chimeras offer an alternative strategy that may exploit the selective dependency observed in TRC-prone cancers.

Finally, combination strategies warrant exploration. KCTD10 inhibition may synergize with agents that exacerbate TRCs or compromise the replication stress response. Careful evaluation of these interactions will be necessary to position KCTD10-targeted therapy optimally within existing treatment paradigms, particularly in tumors already burdened by replication-associated vulnerabilities.

## Data Availability

Not applicable.
